# Knowledge, beliefs, attitude, and practices of E-cigarette use among dental students: A multinational survey

**DOI:** 10.1371/journal.pone.0276191

**Published:** 2022-10-27

**Authors:** Mohammed Nasser Alhajj, Sadeq Ali Al-Maweri, Morenike O. Folayan, Esam Halboub, Yousef Khader, Ridwaan Omar, Abdullah G. Amran, Ola B. Al-Batayneh, Asja Celebić, Sanja Persic, Humeyra Kocaelli, Firas Suleyman, Abdulaziz A. Alkheraif, Darshan D. Divakar, Abdulbaset A. Mufadhal, Mohammed A. Al-Wesabi, Wadhah A. Alhajj, Mokhtar A. Aldumaini, Saadika Khan, Thiyezen A. Al-Dhelai, Ahmed Shaher Alqahtani, Ali H. Murad, Joseph E. Makzoumé, Shivani Kohli, Tareq A. Ziyad

**Affiliations:** 1 Department of Prosthodontics, Faculty of Dentistry, Thamar University, Dhamar, Yemen; 2 College of Dental Medicine, QU Health, Qatar University, Doha, Qatar; 3 Department of Child Dental Health, Obafemi Awolowo University, Ile-Ife, Nigeria; 4 Department of Maxillofacial Surgery and Diagnostic Sciences, College of Dentistry, Jazan University, Jazan, Saudi Arabia; 5 Department of Oral Medicine, Oral Pathology and Oral Radiology, Faculty of Dentistry, Sana’a University, Sana’a, Yemen; 6 Department of Public Health, Community Medicine and Family Medicine, Faculty of Medicine, Jordan University of Science and Technology, Irbid, Jordan; 7 Division of Prosthodontics, Department of Restorative Sciences, Faculty of Dentistry, Kuwait University, Safat, Kuwait; 8 Department of Periodontics, Faculty of Dentistry, Thamar University, Dhamar, Yemen; 9 Department of Preventive Dentistry, Faculty of Dentistry, Jordan University of Science and Technology, Irbid, Jordan; 10 Department of Removable Prosthodontics, School of Dental Medicine and Clinical Hospital Centre, University of Zagreb, Zagreb, Croatia; 11 Department of Oral and Maxillofacial Surgery, Faculty of Dentistry, Istanbul University, Istanbul, Turkey; 12 Department of Prosthodontics, Faculty of Dentistry, University of Health Sciences, İstanbul, Turkey; 13 Dental Biomaterials Research Chair, Dental Health Department, College of Applied Medical Sciences, King Saud University, Riyadh, Kingdom of Saudi Arabia; 14 Department of Conservative Dentistry, Faculty of Dentistry, Sana’a University, Sana’a, Yemen; 15 Department of Preventive and Biomedical Science, Faculty of Dentistry, University of Science and Technology, Taiz, Yemen; 16 Department of Dentistry, Faculty of Medical sciences, Civilization University, Sana’a, Yemen; 17 Department of Fixed and Removable Prosthodontics, Faculty of Dentistry, University of Ibb, Ibb, Yemen; 18 Department of Restorative Dentistry, Faculty of Dentistry, University of the Western Cape, Cape Town, South Africa; 19 Department of Orthodontic and Pediatric Dentistry, College of Dentistry, Qassim University, Buraydah, Saudi Arabia; 20 Department of Orthodontics and Pediatric Dentistry, Faculty of Dentistry, University of Ibb, Ibb, Yemen; 21 Department of Oral Diagnosis, College of Dentistry, University of Al-Qadisiyah, Al-Diwaniya, Iraq; 22 Department of Removable Prosthodontics, Faculty of Dental Medicine, Saint-Joseph University of Beirut, Beirut, Lebanon; 23 Division of Clinical Dentistry, School of Dentistry, International Medical University (IMU), Kuala Lumpur, Malaysia; 24 Private Dental Clinic, Sana’a, Yemen; Xiamen University - Malaysia Campus: Xiamen University - Malaysia, MALAYSIA

## Abstract

**Background:**

E-cigarette use is a trend worldwide nowadays with mounting evidence on associated morbidities and mortality. Dentists can modify the smoking behaviors of their patients. This study aimed to explore the knowledge, beliefs, attitude, and practice of E-cigarette use among dental students.

**Materials and methods:**

This multinational, cross-sectional, questionnaire-based study recruited undergraduate dental students from 20 dental schools in 11 countries. The outcome variable was current smoking status (non-smoker, E-cigarette user only, tobacco cigarette smoker only, dual user). The explanatory variables were country of residence, sex, age, marital status, and educational level. Multiple linear regression analysis was performed to explore the explanatory variables associated with E-cigarette smoking.

**Results:**

Of the 5697 study participants, 5156 (90.8%) had heard about E-cigarette, and social media was the most reported source of information for 33.2% of the participants. For the 5676 current users of E-cigarette and/or tobacco smoking, 4.5% use E-cigarette, and 4.6% were dual users. There were significant associations between knowledge and country (P< 0.05), educational level (B = 0.12; 95% CI: 0.02, 0.21; P = 0.016) and smoking status (P< 0.05). The country of residence (P< 0.05) and smoking status (P< 0.05) were the only statistically significant factors associated with current smoking status. Similarly, there were statistically significant associations between attitude and country (P< 0.05 for one country only compared to the reference) and history of previous E-cigarette exposure (B = -0.52; 95% CI: -0.91, -0.13; P = 0.009). Also, the practice of E-cigarettes was significantly associated with country (P< 0.05 for two countries only compared to the reference) and gender (B = -0.33; 95% CI: -0.52, -0.13; P = 0.001).

**Conclusion:**

The knowledge of dental students about E-cigarette was unsatisfactory, yet their beliefs and attitudes were acceptable. Topics about E-cigarette should be implemented in the dental curriculum.

## Introduction

Electronic cigarettes (E-cigarettes), also known as vaping devices and vape pens, are electronic nicotine delivering systems. These devices deliver nicotine-containing aerosol vapor to a user via heating a liquid (called e-liquid) usually comprised of chemical compounds like propylene glycol, vegetable glycerin, nicotine, and flavorings [1, 2]. The E-cigarette was invented by Hon Lik, a Chinese pharmacist, in 2003 as a habit-breaker for tobacco-cigarette smokers to help users gradually quit smoking [1, 3]. Its global sales have increased dramatically, especially among young adults [4–6], since it got patented in 2007 [1], mainly owing to its promotion as a safer alternative to conventional cigarettes [7]. E-cigarette use among school-aged teens in the USA increased by 78% from 2017 to 2018 [8–12]. There is another device called “heat-not-burn”: It is a new brand of heated tobacco products marketed under the brand name IQOS (I Quit Ordinary Smoking), and uses tobacco leaves, instead of e-liquid, heated at a temperature that does not initiate combustion, and does not contain tartar, although this level of temperature is enough to release a nicotine-containing tobacco vapor which is inhaled by the consumers [13, 14]. The FDA has recently approved this type of product, and some studies suggested that shifting to this type of smoking is still better than continuing with cigarette smoking [15, 16].

E-cigarette use is as huge a concern as the smoking of tobacco. The World Health Organization (WHO) study group on Tobacco Product Regulation recommended subjecting E-cigarette to labeling and warning requirements due to concerns about their impact on health [17]. Due to being a recent habit, the long-term effects of E-cigarette smoking on health have not yet been documented. However, several studies reported its short-term adverse effects [1, 18–20] as listed herein: a case of E-cigarette-related lipoid pneumonia [21], two cases of poisoning [22], traumatic injury resulting from E-cigarette-related explosions [23, 24], and E-cigarette or vaping product use-associated lung injury (EVALI) [25, 26]. Furthermore, many oral health consequences have been reported in association with E-cigarette smoking, such as caries [21], periodontal inflammation [22–24], premature tooth loss [24], xerostomia [27], and softening of the enamel [24]. Based on these consequences, 41 countries have banned the sale of E-cigarette, and 66 countries allow restricted sales [28].

Dental students–future dentists—are expected to play a vital role in the implementation of tobacco cessation programs through educating patients about the hazardous effects of smoking. Therefore, it is essential to know whether E-cigarette use is prevalent in this sector of society and assess their knowledge, beliefs, attitude, and practice in terms of E-cigarette [29]. However, the data on knowledge, beliefs, attitude, and practice of E-cigarette use among dental students have been scarce so far. Based on the health belief model [30], this study was designed to identify knowledge, beliefs, attitude, and practice of E-cigarette use among dental students. We, therefore, postulate that dental students, who are knowledgeable about the harmful effects of E-cigarette, are less likely to indulge in the habit and are less likely to advocate E-cigarette use.

Due to potential variability in cultural and religious norms; availability of tobacco control policies and strategies; as well as advertising, promotion, and marketing efforts of the tobacco industry that can strongly influence the smoking habits of young people, there might be country-wise differences regarding prevalence of E-cigarette use among dental students, and likewise other society segments. Therefore, it is rational and valuable to conduct cross-country and cross-cultural studies on risk indicators for smoking habits. This study sought to determine the differences in knowledge, beliefs, attitude, practices, and source of information about E-cigarette amongst dental students in different countries.

## Methods

### Ethical approval

This study was approved primarily by the Research Ethics Committee at the Faculty of Dentistry, Thamar University, Yemen (Ref#: 2019003). In addition, ethics clearance was obtained from all other participating countries wherever this was a requirement. The study was conducted following the Declaration of Helsinki Ethical Principles.

### Study design, study population and study procedure

We conducted a large-scale, cross-sectional, questionnaire-based project that targeted undergraduate dental students aged 18 years and above in different countries during the academic year 2019/2020. One part of this project (belongs to adverse effects of E-cigarette) has been published elsewhere [31]. The present study adhered strictly to the Strengthening the Reporting of Observational Studies in Epidemiology (STROBE) checklist (S1 Checklist).

The principal investigator sent an email invitation to potential collaborators in 30 countries. Initially, researchers from 25 countries accepted the invitation. Later on, the number of collaborators was reduced to 25 researchers in 20 dental institutes from 11 countries. This was because of the COVID-19 pandemic that resulted in the shut-down of universities and the inability of collaborators in 14 countries to collect data.

Participants were recruited using the snowballing technique. The link to the questionnaire was shared with dental students in the participating institutions using different social media applications, WhatsApp, and emails. The participants were further asked to share the links with their peers.

Before commencing the study, the aims of the study were explained to the participants, and informed consent was obtained. Respondents were instructed to answer the survey once. The questionnaire was flexible to make sure of and edit their answers before they finally submit. Participation was voluntary, and the participants had the right to withdraw from the study at any time without any penalty. By clicking on ‘submit,’ the student consented to participate in the study. Names, emails, or any other personal identifiers were not included in the data collected. Wherever the online survey was not plausible/feasible, a hard copy of the questionnaire was used. No minors were included in the study, and the consent was obtained directly from the participants (not parents or guardians).

### Study instrument

The items of the survey questionnaire were adapted from previously published studies [32–35]. The questionnaire was administered in English using close-ended questions. The full details of the questionnaire are available in an additional file (S1 Questionnaire). A short preface introduced the study team and objectives, assured the confidentiality of the data, and concluded by asking to "agree to participate". In brief, the questionnaire comprised five sections. The first section was about demographic data: age, sex, marital status, and study level (pre-clinical, clinical). In the next part of this section, participants were asked whether they were current smokers or not and whether they had ever heard about E-cigarettes. Only those who responded positively to the latter question (that they had ever heard about E-cigarettes) were asked about their knowledge and beliefs and their source(s) of information about E-cigarettes. The questions on knowledge included: ‘E-cigarettes are harmful to my health’, ‘E-cigarettes reduce passive smoking’, ‘E-cigarettes are less harmful than tobacco cigarettes, ‘E-cigarettes are a better option for my patients than tobacco products’, ‘E-cigarettes are addictive’ and ‘E-cigarettes pose a lower risk of cancer.’

The second section included the following items on participants’ beliefs about E-cigarettes: ‘E-cigarettes help in smoking cessation, ‘E-cigarettes should be banned’ and ‘It is essential for a dentist to be educated about E-cigarettes’. Respondents were also asked for their opinion on the appropriate time to be educated about the harmful effects of E-cigarettes (namely, at school, at university, or no need). Again, this section targeted only those who answered positively to the question of whether that they had ever heard about E-cigarettes.

In the third and fourth sections, tobacco and E-cigarette users were asked about their practices and attitudes. The practice section included questions related to the duration of smoking, how many times they smoke per day, and how soon after waking they start smoking. Attitude questions asked about their confidence to discuss the harmful effects of tobacco or E-cigarette use, their feelings when they smoke, and their attitude about quitting smoking. In the fifth section, E-cigarette-only users were asked about the reasons for initiating E-cigarette use.

#### Outcome and explanatory variables

One of the primary outcome variables of the study was current smoking status (non-smoker, E-cigarette use only, tobacco cigarette smoking only, dual user). The knowledge, beliefs, attitude, and practices were the other primary outcomes. The explanatory variables were country of residence, sex, age, marital status, and educational level.

### Statistical analysis

Data were exported into an Excel Spreadsheet file, double-checked, coded, and then exported to a statistical software program (SPSS V25, IBM Corp., USA) for further analysis. The prevalence of E-cigarette and tobacco smoking was determined. The proportion of the respondents by sex, age, marital status, and educational level were calculated for the 11 countries. The potential associations between these outcome variables and the explanatory variables (country of residence, sex, age, marital status, and educational level) were tested using the Chi-squared test.

For the regression analyses, responses were converted into numerical variables based on the correct answers in the knowledge section or positive responses in the other sections. Then, the total score for each section was calculated (more details about the scoring system are presented in the S1 Table. Accordingly, the bivariate analyses were utilized for differences among the exploratory variables. Variables with significant differences were then entered into the multivariate regression models. Categorical variables with more than 2 sub-categories were converted into dummy variables for more accurate results. A *p*-value less than 0.05 was considered statistically significant.

## Results

A total of 5697 dental students were recruited from 11 countries (Croatia, Iraq, Jordan, Kuwait, Lebanon, Malaysia, Nigeria, Saudi Arabia, South Africa, Turkey, and Yemen). The complete dataset is available in an additional file (S1 Data). The majority of the students were females (60.3%, n = 3433), less than 20-years-old (67.6%, n = 3853), and unmarried (94.1%, n = 5361). Slightly more than half of the subjects (51.1%, n = 2909) were in the clinical years (Table 1). Also, 90.8% (n = 5163) of the students reported that they had heard about E-cigarette, 1215 (23.6%) had tried E-cigarettes before, and 1872 (36.3%) reported that their family members/friends smoke E-cigarettes (S2 Table).

**Table 1:** Current status on usage patterns of E-cigarettes or tobacco cigarettes by participants.

	All participants	Currently smoker (N = 5676)				P
		Never smoke	Tobacco only	E-cigarette only	Dual user	
	5697 (100.0)	4564 (80.4)	596 (10.5)	255 (4.5)	261 (4.6)	
**Country**						
Croatia	233 (4.1)	175 (75.1)	49 (21.0)	5 (2.1)	4 (1.7)	<0.001
Iraq	369 (6.5)	300 (81.3)	31 (8.4)	11 (3.0)	27 (7.3)	
Jordan	461 (8.1)	343 (74.4)	45 (9.8)	44 (9.5)	29 (6.3)	
Kuwait	110 (1.9)	103 (93.6)	0 (0.0)	7 (6.4)	0 (0.0)	
Lebanon	257 (4.5)	220 (85.6)	27 (10.5)	4 (1.6)	6 (2.3)	
Malaysia	148 (2.6)	139 (93.9)	1 (0.7)	3 (2.0)	5 (3.4)	
Nigeria	240 (4.2)	228 (95.0)	7 (2.9)	5 (2.1)	0 (0.0)	
Saudi Arabia	596 (10.5)	430 (72.6)	55 (9.3)	69 (11.7)	38 (6.4)	
South Africa	204 (3.6)	177 (86.8)	16 (7.8)	7 (3.4)	4 (2.0)	
Turkey	1453 (25.5)	1090 (75.0)	208 (14.3)	59 (4.1)	96 (6.6)	
Yemen	1626 (28.5)	1359 (84.5)	157 (9.8)	41 (2.5)	52 (3.2)	
**Gender**						
Male	2264 (39.7)	1594 (70.6)	344 (15.2)	176 (7.8)	144 (6.4)	<0.001
Female	3433 (60.3)	2970 (86.9)	252 (7.4)	79 (2.3)	117 (3.4)	
**Age group**						
≤ 20 years	≤ 20 years	1502 (81.7)	178 (9.7)	72 (3.9)	86 (4.7)	0.227
> 20 years	> 20 years	3062 (79.8)	418 (10.9)	183 (4.8)	175 (4.6)	
**Marital status**						
Married	336 (5.9)	259 (77.1)	40 (11.9)	13 (3.9)	24 (7.1)	0.090
Unmarried	5361 (94.1)	4305 (80.6)	556 (10.4)	242 (4.5)	237 (4.4)	
**Educational level**						
Pre-clinical	2788 (48.9)	2242 (80.8)	295 (10.6)	105 (3.8)	134 (4.8)	0.076
Clinical	2909 (51.1)	2322 (80.1)	301 (10.4)	150 (5.2)	127 (4.4)	

P-value < 0.05 is considered significant.

The current status on the use of E-cigarette and/or tobacco smoking (N = 5676) are presented in Table 1. The majority of students (n = 4564, 80.4%) reported they never smoked, while 1112 (19.6%) students reported they were current smokers; of whom 596 (10.5%) students smoke tobacco only, 255 (4.5%) students use E-cigarettes only, and 261 (4.7%) students were dual users. There were statistically significant gender-wise and country-wise differences regarding the use of E-cigarettes and/or tobacco cigarettes (*P*< 0.01). Meanwhile, no significant associations were found between smoking status and marital status, age, or level of study (Table 1).

The proportions of students who gave correct responses to the different E-cigarettes knowledge questions ranged from 22.8% and 78.4% (Table 2). The majority (78.4%, n = 4035) of the students reported that E-cigarettes are harmful to health, 2160 (42%) reported that E-cigarettes pose a high risk for cancer, 2673 (51.9%) reported that E-cigarettes are addictive, 1148 (22.8%) reported that The FDA does not approve e-cigarettes, and 1833 (35.8%) reported that E-cigarettes do not reduce passive smoking. Interestingly, current E-cigarette users revealed poorer knowledge about E-cigarettes than non-smokers and tobacco cigarette users did (*P*< 0.01).

**Table 2:** Knowledge and beliefs about E-cigarettes (N = 5163).

	All participants	Currently smoker				P
		Never smoke	Tobacco only	E-cigarette only	Dual user	
**Knowledge about E-cigarette; correct answer reported**						
**E-cigarettes are approved by the FDA**	1148 (22.8)	818 (71.3)	163 (14.2)	94 (8.2)	73 (6.4)	<0.001
**E-cigarettes are harmful to my health**	4035 (78.4)	3332 (82.6)	392 (9.7)	156 (3.9)	155 (3.8)	<0.001
**E-cigarettes reduce passive smoking**	1833 (35.8)	1523 (83.1)	190 (10.4)	45 (2.5)	75 (4.1)	<0.001
**E-cigarettes are less harmful than tobacco cigarette**	1703 (33.1)	1412 (82.9)	191 (11.2)	37 (2.2)	63 (3.7)	<0.001
**E-cigarettes are a better option for my patients than tobacco products**	1252 (24.3)	996 (79.6)	165 (13.2)	34 (2.7)	57 (4.6)	<0.001
**E-cigarettes are addictive**	2673 (51.9)	2228 (83.4)	255 (9.5)	95 (3.6)	95 (3.6)	<0.001
**E-cigarettes pose a lower risk of cancer**	2160 (42.0)	1857 (86.0)	195 (9.0)	38 (1.8)	70 (3.2)	<0.001
**Beliefs about E-cigarette; positive answer is reported**						
**E-cigarettes help with smoking cessation**	1625 (31.6)	1123 (69.1)	190 (11.7)	180 (11.1)	132 (8.1)	<0.001
**It is essential for a dentist to be educated about E-cigarettes**	4487 (87.2)	3621 (80.7)	457 (10.2)	210 (4.7)	199 (4.4)	<0.001
**E-cigarettes should be banned**	2451 (47.8)	2161 (88.2)	179 (7.3)	49 (2.0)	62 (2.5)	<0.001
**What is the right time to be educated about harmful effects of E-cigarettes**	3929 (76.8)	3167 (80.6)	411 (10.5)	175 (4.5)	176 (4.5)	<0.001

P-value < 0.05 is considered significant.

Around one-third (30.7%) of the students believed that E-cigarettes help in tobacco cessation. The majority (n = 4487, 87.2%) believed that dental practitioners should be educated about E-cigarettes, and 2451 (47.8%) agreed that E-cigarettes should be banned. In addition, 3929 (76.8%) students believed that education about E-cigarettes should start during the school years. Again, E-cigarette users significantly had more negative beliefs about E-cigarettes when compared to that of non-smokers and tobacco cigarette smokers (*P*< 0.01; Table 2).

**Fig 1:**
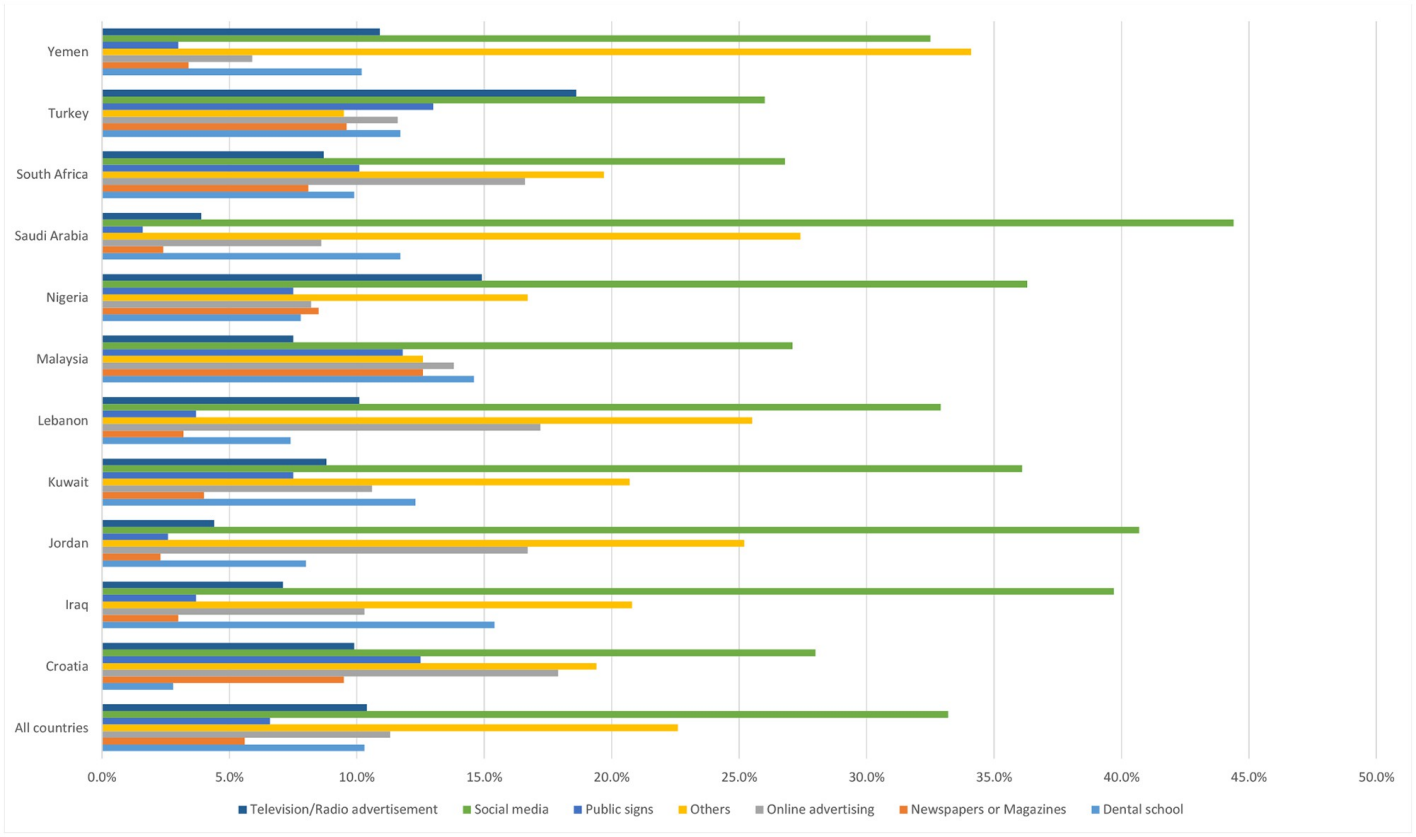
Source of information about E-cigarette.

The sources of information about E-cigarette were social media (33.2%), online advertising (11.3%), dental school (10.3%), television/radio (10.4%), public signs (6.6%) and others (22.6%), and these sources differed significantly by country (Fig 1).

The responses to the items of E-cigarette practice and attitudes are presented in Table 3. The majority of smokers (all types of smoking) reported that they had been smoking for more than 2 years and for less than 20 times a day. In contrast, the majority of E-cigarette users reported that they had been vaping for less than 1 year and for less than 20 times a day (Table 3). In addition, 734 (66.1%) smokers reported that they feel confident to discuss the harmful effects of tobacco use; no significant differences were found between tobacco and E-cigarette users. In contrast, 534 (48.1%) reported that they feel confident to discuss the harmful effects of E-cigarettes, with significant differences between tobacco and E-cigarette users (47.8% vs. 27.0%, *P* = 0.003). Further, 57.7% of the tobacco users, 21.7% of E-cigarette users, and 20.6% of dual smokers reported their wishes to quit their habit.

**Table 3:** E-cigarette use and tobacco smoking practice and attitude by participants (N = 1112).

	All participants	Currently smoker			P
		Tobacco only	E-cigarette only	Dual user	
**Practice of E-cigarette and tobacco smoking**					
Duration of smoking					
< 1 year	324 (35.2)	141 (43.5)	107 (33.0)	76 (23.5)	<0.001
1–2 years	217 (23.6)	114 (52.5)	58 (26.7)	45 (20.7)	
> 2 years	379 (41.2)	241 (63.6)	59 (15.6)	79 (20.8)	
Smoking per day					
Not daily	314 (33.9)	184 (58.6)	72 (22.9)	58 (18.5)	0.064
< 20 times a day	443 (47.9)	232 (52.4)	104 (23.5)	107 (24.2)	
≥ 20 times a day	168 (18.2)	78 (46.4)	51 (30.4)	39 (23.2)	
When start smoking					
Immediately After wake-up	163 (17.9)	76 (46.6)	50 (30.7)	37 (22.7)	0.287
After 1–2 hours	286 (31.5)	150 (52.4)	70 (24.5)	66 (23.1)	
It varies	460 (50.6)	256 (55.7)	106 (23.0)	98 (21.3)	
**Attitude about E-cigarette and tobacco smoking; positive answer is reported**					
Feeling confident to discuss harmful effects of tobacco cigarettes	734 (66.1)	395 (53.8)	181 (24.7)	158 (21.5)	0.173
Feeling confident to discuss harmful effects of E-cigarettes	534 (48.1)	255 (47.8)	144 (27.0)	135 (25.3)	0.003
Superiority among friends	501 (53.5)	278 (55.5)	127 (25.3)	96 (19.2)	0.156
Feel more pleasure	231 (24.9)	127 (55.0)	52 (22.5)	52 (22.5)	0.460
Relieve stress	190 (20.4)	99 (52.1)	41 (21.6)	50 (26.3)	0.067
I want to quit smoking	525 (57.1)	303 (57.7)	114 (21.7)	108 (20.6)	0.002

P-value < 0.05 is considered significant.

**Fig 2:**
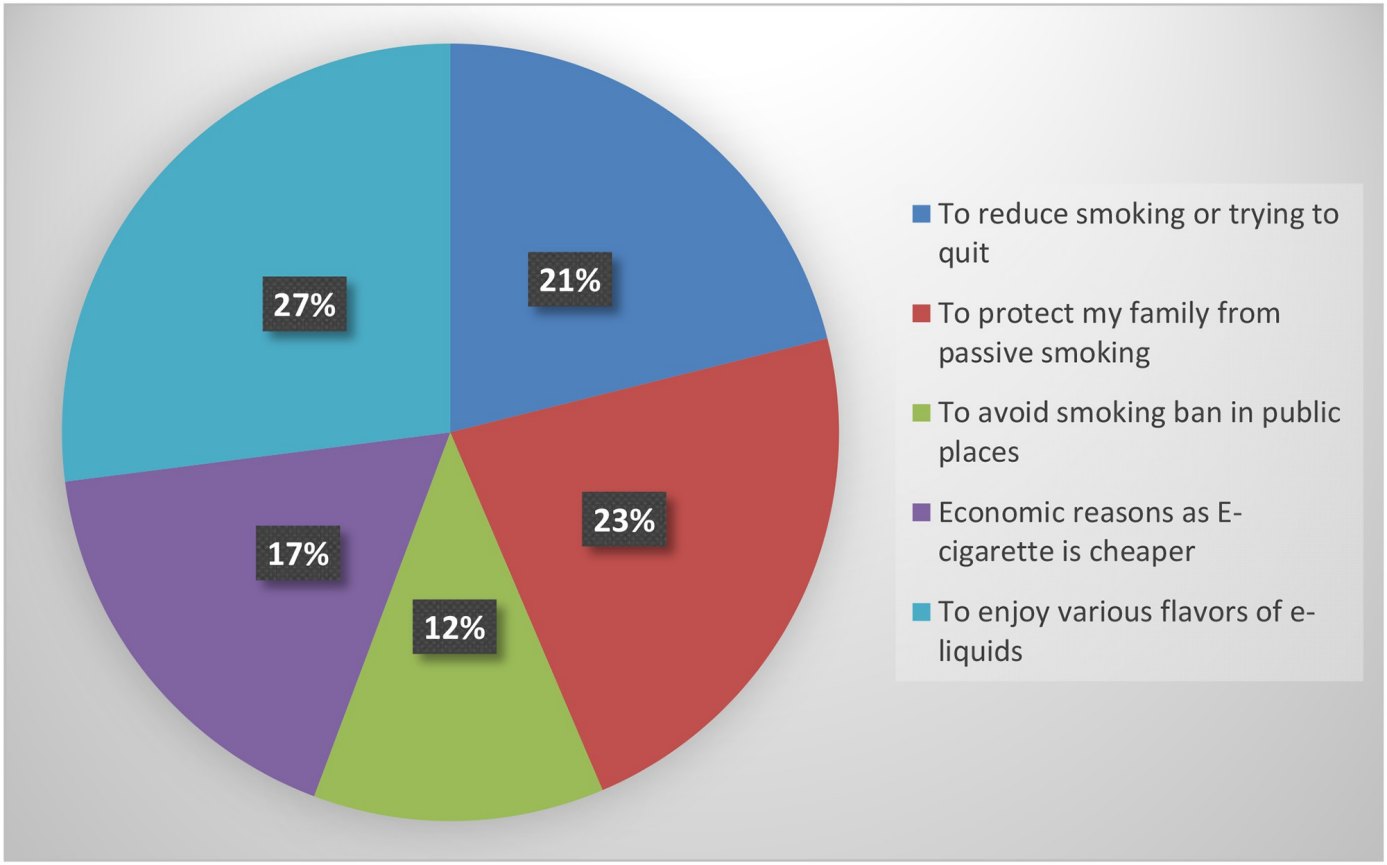
Reason(s) for initiating use of E-cigarette (E-cigarette smokers); important reason is reported.

The most cited reasons were the desire to enjoy the flavors of E-liquids (27%) followed by protection of the family from passive smoking (23%) and quitting tobacco (21%). More details about the reasons for initiating E-cigarettes are shown in Fig 2.

The findings on the factors associated with the overall score of knowledge, beliefs, attitudes, and practice are presented in the S3 and S4 Tables. In brief, there were significant associations between knowledge and some explanatory variables, namely: country (highest in Malaysia), gender (higher for female), training stage (higher for clinical years), never having tried E-cigarette, and smoking status (highest for never smokers). Positive beliefs were significantly associated with the country (highest in Jordan and Kuwait), gender (higher for females), marital status (higher for married students), never having tried E-cigarette and never smokers (highest for never smokers). There were significant associations of attitude with country, gender, age group, academic level, and history of smoking (P< 0.05 each). There were also significant associations of practice with country, gender, and type of smoke (P< 0.01 each).

Results of the multiple linear regression analyses are presented in Table 4. There were significant associations between knowledge and country (P< 0.05), educational level (B = 0.12; 95% CI: 0.02, 0.21; P = 0.016) and smoking status (P< 0.05). Regarding beliefs, country (P< 0.05) and smoking status (P< 0.05) were the only statistically significant independent factors. Similarly, there were statistically significant associations between attitude and country (P< 0.05 for one country only compared to the reference) and history of previous E-cigarette exposure (B = -0.52; 95% CI: -0.91, -0.13; P = 0.009). Also, the practice of E-cigarettes was significantly associated with country (P< 0.05 for two countries only compared to the reference) and gender (B = -0.33; 95% CI: -0.52, -0.13; P = 0.001).

**Table 4:** Factors associated with knowledge, beliefs, attitude, and practice of E-cigarettes.

		Knowledge (N = 5163)				Beliefs (N = 5163)				Attitude (N = 1112)				Practice (N = 1112)			
		B	95.0% CI for B		P	B	95.0% CI for B		P	B	95% CI for B		P	B	95% CI for B		P
			Lower	Upper			Lower	Upper			Lower	Upper			Lower	Upper	
**Country**	Croatia	-1.03	-1.39	-0.68	<0.001	-0.66	-0.86	-0.47	<0.001	-0.65	-2.65	1.35	0.525	-0.58	-1.78	0.63	0.349
	Iraq	-0.95	-1.29	-0.62	<0.001	-0.44	-0.61	-0.26	<0.001	-0.68	-2.66	1.31	0.504	-0.87	-2.06	0.32	0.152
	Jordan	-0.65	-0.97	-0.32	<0.001	Reference				-0.36	-2.28	1.57	0.717	-0.89	-2.06	0.27	0.134
	Kuwait	-0.72	-1.14	-0.3	0.001	-0.13	-0.39	0.12	0.308	Reference				-1.25	-2.81	0.3	0.114
	Lebanon	-1.15	-1.51	-0.8	<0.001	-0.57	-0.77	-0.38	<0.001	-0.87	-2.94	1.2	0.409	-0.64	-1.9	0.62	0.32
	Malaysia	Reference				-0.34	-0.57	-0.11	0.003	-1.13	-3.62	1.36	0.373	Reference			
	Nigeria	-1.21	-1.59	-0.83	<0.001	-0.24	-0.46	-0.01	0.038	-1.29	-3.78	1.2	0.309	-1.57	-3.18	0.04	0.055
	Saudi Arabia	-0.92	-1.23	-0.6	<0.001	-0.42	-0.57	-0.26	<0.001	-0.65	-2.56	1.26	0.507	-0.99	-2.15	0.17	0.095
	South Africa	-0.89	-1.26	-0.52	<0.001	-0.42	-0.63	-0.22	<0.001	-0.54	-2.64	1.56	0.616	-0.45	-1.73	0.83	0.492
	Turkey	-0.24	-0.54	0.05	0.105	-0.94	-1.08	-0.81	<0.001	-2.34	-4.26	-0.42	0.017	-1.35	-2.5	-0.2	0.021
	Yemen	-0.81	-1.11	-0.52	<0.001	-0.31	-0.45	-0.18	<0.001	-0.57	-2.48	1.35	0.561	-1.22	-2.38	-0.06	0.04
**Gender**	Male	0.06	-0.04	0.16	0.244	0.06	-0.01	0.13	0.104	-0.18	-0.5	0.15	0.289	-0.33	-0.52	-0.13	0.001
	Female	Reference				Reference				Reference				Reference			
**Educational level**	Pre-clinical	0.12	0.02	0.21	0.016												
	Clinical	Reference															
**Marital status**	Married					-0.1	-0.24	0.05	0.196								
	Unmarried					Reference											
**Age groups**	≤ 20 years									-0.11	-0.46	0.25	0.553				
	> 20 years									Reference							
**Tried E-cigarette**	Yes	0.07	-0.07	0.21	0.341	0.05	-0.05	0.15	0.338	-0.52	-0.91	-0.13	0.009				
	No	Reference				Reference				Reference							
**Family/friends smoke E-cig.**	Yes	-0.03	-0.14	0.08	0.574	0.04	-0.04	0.12	0.287	0.13	-0.24	0.49	0.501				
	No	Reference				Reference				Reference							
**Currently smoker**	T-cig. only	-0.22	-0.38	-0.06	0.007	-0.25	-0.37	-0.14	0	0.15	-0.28	0.58	0.487	Reference			
	E-cig. only	-0.96	-1.21	-0.72	<0.001	-0.14	-0.32	0.03	0.113	Reference				-0.18	-0.42	0.05	0.128
	Dual user	-0.63	-0.87	-0.39	<0.001	-0.28	-0.45	-0.1	0.002	-0.27	-0.73	0.18	0.24	0.21	-0.03	0.45	0.083
	Never smoke	Reference				Reference											

P-value < 0.05 is considered significant.

## Discussion

The present large-scale multicounty survey assessed the knowledge, beliefs, attitude, and practice of E-cigarette use among dental students across 11 countries. Generally, the results revealed that surveyed dental students have relatively positive beliefs and attitudes towards E-cigarette, but, unfortunately, unsatisfactory levels of knowledge with some significant differences across countries. Additionally, the results revealed that most students were non-smokers, while only 4.5% of students were E-cigarette users and 4.7% of them were dual users.

The surveyed students in the present study showed generally poor knowledge about E-cigarettes (overall score 2.9 out of 7). A study conducted in Saudi Arabia reported the level of knowledge among dental students regarding approval of E-cigarettes by the FDA as 28%, regarding posing a lower risk for cancer as 52%, and regarding the question whether E-cigarettes are a better option for dental patients than tobacco smoking as 21% [36]. Correspondingly, 4% and 39% of US medical students thought that E-cigarette use is approved by FDA and reduces the risk of cancer, respectively [34]. Surprisingly, in the present study, smokers had the lowest level of knowledge on questions about FDA approval and cancer risk, which raises many questions as to whether this reflects the respondent’s real knowledge, given that they are dental students, or whether as smokers they are trying to evade the truth and convince themselves that smoking (including E-cigarettes) is safe. Both scenarios would be extremely unfortunate given that such dental students are very likely to become bad role models for their patients in the near future. The levels of knowledge shown by Malaysian and Turkish students were statistically higher than those shown by students of the other countries, which is not easy to explain. However, in Malaysia, smoking cessation is taught to dental students as a part of the dental curriculum in some universities. It is introduced as early as year 2 and it continues till year 5, where it is reinforced regularly during students’ clinical practice. In a recent qualitative study, college students in the US revealed an acceptable knowledge on addictiveness to E-cigarettes but poorer low knowledge on its harmful effects on health [37]. Conversely, 96.5% [6] and 85.6% [34] of medical students in the US thought that E-cigarette use is harmful to health, and is addictive, respectively.

Further, 51.7% of Saudi students thought that E-cigarettes are less addictive than tobacco smoking, but only 29.8% of these students thought that E-cigarettes are a source of second-hand exposure to nicotine [38]. The corresponding proportions in our study were 51.9% and 64.2%, respectively. Interestingly, a recent study showed that a large proportion of US adults perceived E-cigarettes as equally or more harmful than cigarettes, and this proportion has increased substantially from 2012 to 2015 [39] and from 2012 to 2017 [40]. In the Asia-Pacific study, a sizable number of E-cigarette users reported that E-cigarettes were less harmful than combustible cigarettes to both the smoker (45.1%) and to people around them (46.2%) [41].

Driven by the critical importance of having a high level of knowledge on E-cigarettes, Briggs et al., after surveying dental professionals, dental associations, and regulatory authorities, presented updated information about E-cigarettes, its harmful effects, counseling advice, and proposed regulations [42]. The internet, represented mostly by social media, is unsurprisingly the main source of information about many daily matters, including E-cigarettes (33%), while dental schools paradoxically do not contribute more than 10%. The former is not surprising as everyone is immersed in the internet, and it has become an integral part of our lives. Many similar studies support our results [34, 43–45]. Moreover, a new content-analysis study [46] found that the term “vape” in the headline and internet contents was not frequently associated with the term “cigarette,” which may reduce the negative perception of tobacco use, making it more pleasing.

For the present group of responders, notwithstanding their relatively low levels of knowledge, especially among smokers and the E-cigarette users and dual users, the belief was still somewhat acceptable (overall score 5.8 out 8). Similarly, the attitude was acceptable (6 out of 9). The scores of the beliefs and attitudes fluctuated correspondingly with the scores of knowledge: more negative overall scores were obtained by E-cigarette users and dual users. Studies on the beliefs and attitudes of dental or other college students with regard to E-cigarettes have been scarce and have generally reported conflicting results. In contrast to the 47.8% in our study, the Asia-Pacific study showed that 71.6% of university students supported government regulation of E-cigarettes [41], while 92% of dental students from Italy and Poland support a ban of E-cigarettes [47]. Similar to the current study, Italian and Polish dental students reported willingness to quit smoking [47].

In contrast to our study, Saudi dental students reported that they feel confident to discuss the harmful effects of E-cigarettes and the importance of a dentist being educated about E-cigarettes, respectively, 31, which indicates a more positive attitude in our sample. In line with that, 12.3% of medical students from the US feel confident to discuss the harmful effects of E-cigarettes with patients [34]. Up to 59% and 28% of a sample of Saudi medical students thought that E-cigarettes help smoking cessation and will recommend their use to their patients, respectively [48]. In a recent study, French laypersons showed a similar level of positive beliefs with regard to the item “E-cigarettes help quit smoking” [49]. Compared to 31.6% in our study, a study from Egypt reported that up to 13% and 20% of healthcare providers and the general population, respectively, thought that E-cigarette use is helpful for smoking cessation [50]. It is worth noting that E-cigarette users and dual users reported more negative beliefs/attitudes compared to tobacco smokers, and to a larger extent, to never smokers. This is surprising and difficult to explain given that all are dental students. However, the above-mentioned variations in knowledge may help to explain the results, quite beside the fact that the variability in knowledge itself needs to be better understood and explained.

Perhaps indicative of being addicted to E-cigarette use, most E-cigarette users and dual users in our study smoke immediately upon, or 1–2 hours after, waking up, while a small proportion of them smoke more than 20 times/day. Interestingly, only 3.6% of them considered E-cigarette use addictive in comparison to 9.5% and 83.4% of tobacco smokers and never smokers, respectively. Indeed, in a recent large study participants scored E-cigarettes as addictive as alcohol (5.61/8 versus 5.49/8) and more addictive than Marijuana (4.81/8) [43]. Sizable proportions of E-cigarette users and dual users in the current study indulged in the habit recently (< 1 year to 2 years) in comparison to the tobacco smokers, supporting the fact that E-cigarette use is a recent trend.

Around 27%, 23%, and 21% of E-cigarette users in the current study reported that they do so to enjoy E-cigarettes’ flavors, protect their families from passive smoking, and reduce/quit smoking, respectively. Somewhat comparable proportions were reported elsewhere [45, 51–53]. This may further explain the extent of the addictiveness of smoking habits, with users trying to find alternatives rather than quit. Many studies reported other reasons for indulging in E-cigarette use, including, among others: being the current trend [45, 52]; and it being used by friends and/or family members [51, 52]. The former can be explained as being driven by the marketing advertisements nowadays are even more widely penetrating owing to the internet. At the same time, the latter can be attributed to the influence of the role model of friends and/or family members.

The multivariable analyses revealed the “country” variable as an independent determinant of knowledge and beliefs in favor of Malaysia (and Turkey to a lesser extent) than other countries. The situation is completely different as regards attitudes and practices, with no country-wise variability being evident. This further complicates how to justify the gap between “what is known” and “what is being done”. Contrary to what is expected, it would appear that the lower the educational level of the participants, the higher their knowledge on E-cigarettes. It would seem self-evident that with exposure to further information upon progressing to higher educational levels, there would be a higher level of knowledge. It may be speculated that such a strange and surprising result might be attributed to the stress that students face with the start of the clinical work at higher levels [54, 55]. The tobacco cigarettes and E-cigarettes serve to relieve stress. Another independent determinant of knowledge and beliefs was smoking status: “never-smokers” reflected a higher level of knowledge and positive beliefs than smokers. This is further substantiated by the results of attitudes and practices that were not significantly different among cigarette smokers, E-cigarette users, and dual users. What reinforces this result further was the significant negative attitude among those who tried E-cigarettes compared to those who had not. There was no significant difference gender-wise except for practice in which females reflected more positive behaviors than males. Generally, it may be said that females indulge in bad habits less frequently than do males despite their comparable knowledge, attitude, and belief; and this might be due to many factors including, but not limited to, traditions, culture, and religion. Finally, although the COVID-19 pandemic had great psychological and economic negative effects on the public, a new study [56] among youths and young adults revealed that the “stay-at-home” directives were associated with a decline in E-cigarette use among these groups of people.

### Study limitations

Despite being a multinational study, many limitations should be highlighted. First, the snowballing recruitment technique may have skewed the sample a bit if students shared it with friends who had similar demographics and lifestyles. A second limitation is the cross-sectional nature of the study and using a self-administered questionnaire, a tool that is criticized due to recall bias and subjectivity. Thirdly, as data were collected online, this may inadvertently had excluded students who did not have internet access. This excluded population may represent students with lower socioeconomic status, a matter which further may bias the obtained results. Fourthly, the included countries were composed of developed and developing countries, reflecting different cultures, and different education and health systems, a matter which also may contaminate the results and make the explanation challenging. Finally, the study did not explore the factors that prompt the use of tobacco and E-cigarettes by dental students; this may help to facilitate the design of programs to prevent or break the habit.

### Study implications

Based on the proverb states “who does not have thing cannot give it”, our results put doubts on the ability of dental students -at least in the included countries- of giving cessation counselling advice to their patients. Dental students are the future dentists, and hence they must behave as role models, otherwise their message in community service will be largely jeopardized. Accordingly, dental curriculum in the institutions from the included countries must be reshaped in a manner that implants positive knowledge and beliefs, which later on grow into positive behavior. Doing so guarantees growing of a positive conviction among dental students to be future role models to their communities.

To our knowledge, this study provides a report on multiple aspects of E-cigarettes use in countries where data on this habit is limited. The study findings indicate that for dental students to be able to able to provide tobacco cessation counselling, there may be the need to proactively include information on tobacco and |E-cigarette smoking in the education curriculum of dental students in the countries included in the survey.

#### Recommendation and future direction

Further well-designed, large scale studies with emphasis to include more countries and representative samples are highly encouraged. Including objective outcomes (biological parameters) must be stressed on. Moreover, similar studies addressing other health students: medical, nursing and so on are recommended. While planning for curriculum changes, pre- and post- studies will be helpful regarding the desired effects, and modifications if necessary.

## Conclusion

The prevalence of E-cigarette use among dental students is considered high. The overall knowledge about E-cigarette among dental students is unsatisfactory. Overall, beliefs and attitudes of dental students are acceptable, but their practice is considered poor. However, differences by country, smoking status, and gender were evident. In general, well-designed, large-scale studies with larger sample sizes covering more countries are to be encouraged. Education on the adverse effects of E-cigarette smoking must be included in the dental curricula, so the students can realize these adverse effects and be good models for their future patients.

## Supporting information

S1 Checklist STROBE checklist. (DOCX)

S1 Questionnaire E-cigarette questionnaire. (DOCX)

S1 Data E-Cigarette survey dataset. (XLSX)

S1 Table Scoring system of the study variables. (DOCX)

S2 Table Characteristics of participants by country. (DOCX)

S3 Table Bivariate association between knowledge, beliefs and study variables. (DOCX)

S4 Table Bivariate association between attitude, practice and study variables. (DOCX)

## Abbreviations

COVID: Coronavirus Disease
E-cigarette: Electronic cigarettes
EVALI: E-cigarette or vaping product use-associated lung injury
FDA: Food and Drug Administration
IQOS: I Quit Ordinary Smoking
SPSS: Statistical Package for the Social Sciences
US: United States
WHO: World Health Organization
