# Supplementary material for: Knowledge, beliefs, attitude, and practices of E-cigarette use among dental students: A multinational survey
Source: PLoS One. 2022 Oct 27;17(10):e0276191. doi: 10.1371/journal.pone.0276191 (PMC9612543; doi:10.1371/journal.pone.0276191)
Supplement: S1 Checklist — (DOCX) [file pone.0276191.s001.docx]

STROBE Statement—checklist of items that should be included in reports of observational studies

|  | Item No. | Recommendation | Page  No. | Relevant text from manuscript |
| --- | --- | --- | --- | --- |
| **Title and abstract** | 1 | (*a*) Indicate the study’s design with a commonly used term in the title or the abstract | 1 | Knowledge, Beliefs, Attitude, and Practices of E-cigarette Use among Dental Students: A Multinational Survey |
|  |  | (*b*) Provide in the abstract an informative and balanced summary of what was done and what was found | 3 | This multinational, cross-sectional, questionnaire-based study recruited undergraduate dental students from 20 dental schools in 11 countries. The outcome variable was current smoking status (non-smoker, E-cigarette user only, tobacco cigarette smoker only, dual user). The explanatory variables were country of residence, sex, age, marital status, and educational level. Multiple linear regression analysis was performed to explore the explanatory variables associated with E-cigarette smoking. **Results:** Of the 5697 study participants, 5156 (90.8%) had heard about E-cigarette, and social media was the most reported source of information for 33.2% of the participants. For the 5676 current users of E-cigarette and/or tobacco smoking, 4.5% use E-cigarette, and 4.6% were dual users. There were significant associations between knowledge and country (P< 0.05), educational level (B= 0.12; 95% CI: 0.02, 0.21; P=0.016) and smoking status (P< 0.05). The country of residence (P< 0.05) and smoking status (P< 0.05) were the only statistically significant factors associated with current smoking status. Similarly, there were statistically significant associations between attitude and country (P< 0.05 for one country only compared to the reference) and history of previous E-cigarette exposure (B= -0.52; 95% CI: -0.91, -0.13; P= 0.009). Also, the practice of E-cigarettes was significantly associated with country (P< 0.05 for two countries only compared to the reference) and gender (B= -0.33; 95% CI: -0.52, -0.13; P= 0.001). |
| Introduction | | | |  |
| Background/rationale | 2 | Explain the scientific background and rationale for the investigation being reported | 5 | Dental students – future dentists - are expected to play a vital role in the implementation of tobacco cessation programs through educating patients about the hazardous effects of smoking. Therefore, it is essential to know whether E-cigarette use is prevalent in this sector of society and assess their knowledge, beliefs, attitude, and practice in terms of E-cigarette. However, the data on knowledge, beliefs, attitude, and practice of E-cigarette use among dental students have been scarce so far. Based on the health belief model |
| Objectives | 3 | State specific objectives, including any prespecified hypotheses | 5 | To identify knowledge, beliefs, attitude, and practice of E-cigarette use among dental students. We, therefore, postulate that dental students, who are knowledgeable about the harmful effects of E-cigarette, are less likely to indulge in the habit and are less likely to advocate E-cigarette use. |
| Methods | | | |  |
| Study design | 4 | Present key elements of study design early in the paper | 5 | We conducted a large-scale, cross-sectional, questionnaire-based project that targeted undergraduate dental students aged 18 years and above |
| Setting | 5 | Describe the setting, locations, and relevant dates, including periods of recruitment, exposure, follow-up, and data collection | 6 | The study targeted undergraduate dental students aged 18 years and above in different countries during the academic year 2019/2020. One part of this project (belongs to adverse effects of E-cigarette) has been published elsewhere [31].  The principal investigator sent an email invitation to potential collaborators in 30 countries. Initially, researchers from 25 countries accepted the invitation. Later on, the number of collaborators was reduced to 25 researchers in 20 dental institutes from 11 countries. This was because of the COVID-19 pandemic that resulted in the shut-down of universities and the inability of collaborators in 14 countries to collect data.  Participants were recruited using the snowballing technique. The link to the questionnaire was shared with dental students in the participating institutions using different social media applications, WhatsApp, and emails. The participants were further asked to share the links with their peers. |
| Participants | 6 | (*a*) *Cohort study*—Give the eligibility criteria, and the sources and methods of selection of participants. Describe methods of follow-up  *Case-control study*—Give the eligibility criteria, and the sources and methods of case ascertainment and control selection. Give the rationale for the choice of cases and controls  *Cross-sectional study*—Give the eligibility criteria, and the sources and methods of selection of participants | 6 | The study targeted undergraduate dental students aged 18 years and above in different countries during the academic year 2019/2020. |
|  |  | (*b*) *Cohort study*—For matched studies, give matching criteria and number of exposed and unexposed  *Case-control study*—For matched studies, give matching criteria and the number of controls per case |  |  |
| Variables | 7 | Clearly define all outcomes, exposures, predictors, potential confounders, and effect modifiers. Give diagnostic criteria, if applicable | 7 | One of the primary outcome variables of the study was current smoking status (non-smoker, E-cigarette use only, tobacco cigarette smoking only, dual user). The knowledge, beliefs, attitude, and practices were the other primary outcomes. The explanatory variables were country of residence, sex, age, marital status, and educational level. |
| Data sources/ measurement | 8* | For each variable of interest, give sources of data and details of methods of assessment (measurement). Describe comparability of assessment methods if there is more than one group | *6, 7* | *The items of the survey questionnaire were adapted from previously published studies [32-35]. The questionnaire was administered in English using close-ended questions. A short preface introduced the study team and objectives, assured the confidentiality of the data, and concluded by asking to "agree to participate". In brief, the questionnaire comprised five sections. The first section was about demographic data: age, sex, marital status, and study level (pre-clinical, clinical). In the next part of this section, participants were asked whether they were current smokers or not and whether they had ever heard about E-cigarettes. Only those who responded positively to the latter question (that they had ever heard about E-cigarettes) were asked about their knowledge and beliefs and their source(s) of information about E-cigarettes. The questions on knowledge included: ‘E-cigarettes are harmful to my health’, ‘E-cigarettes reduce passive smoking’, ‘E-cigarettes are less harmful than tobacco cigarettes, ‘E-cigarettes are a better option for my patients than tobacco products’, ‘E-cigarettes are addictive’ and ‘E-cigarettes pose a lower risk of cancer.’*  *The second section included the following items on participants’ beliefs about E-cigarettes: ‘E-cigarettes help in smoking cessation, ‘E-cigarettes should be banned’ and ‘It is essential for a dentist to be educated about E-cigarettes’. Respondents were also asked for their opinion on the appropriate time to be educated about the harmful effects of E-cigarettes (namely, at school, at university, or no need). Again, this section targeted only those who answered positively to the question of whether that they had ever heard about E-cigarettes.*  *In the third and fourth sections, tobacco and E-cigarette users were asked about their practices and attitudes. The practice section included questions related to the duration of smoking, how many times they smoke per day, and how soon after waking they start smoking. Attitude questions asked about their confidence to discuss the harmful effects of tobacco or E-cigarette use, their feelings when they smoke, and their attitude about quitting smoking. In the fifth section, E-cigarette-only users were asked about the reasons for initiating E-cigarette use.* |
| Bias | 9 | Describe any efforts to address potential sources of bias |  | NA |
| Study size | 10 | Explain how the study size was arrived at |  | NA |
| Continued on next page Quantitative variables | 11 | Explain how quantitative variables were handled in the analyses. If applicable, describe which groupings were chosen and why | 7, 8 |  |
| Statistical methods | 12 | (*a*) Describe all statistical methods, including those used to control for confounding | 8 | The potential associations between these outcome variables and the explanatory variables (country of residence, sex, age, marital status, and educational level) were tested using the Chi-squared test.  For the regression analyses, responses were converted into numerical variables based on the correct answers in the knowledge section or positive responses in the other sections. Then, the total score for each section was calculated (more details about the scoring system are presented in the Supplementary file, Table 1). Accordingly, the bivariate analyses were utilized for differences among the exploratory variables. Variables with significant differences were then entered into the multivariate regression models. Categorical variables with more than 2 sub-categories were converted into dummy variables for more accurate results. A *p*-value less than 0.05 was considered statistically significant. |
|  |  | (*b*) Describe any methods used to examine subgroups and interactions |  | Categorical variables with more than 2 sub-categories were converted into dummy variables for more accurate results. |
|  |  | (*c*) Explain how missing data were addressed | NA |  |
|  |  | (*d*) *Cohort study*—If applicable, explain how loss to follow-up was addressed  *Case-control study*—If applicable, explain how matching of cases and controls was addressed  *Cross-sectional study*—If applicable, describe analytical methods taking account of sampling strategy | NA |  |
|  |  | (*e*) Describe any sensitivity analyses | NA |  |
| Results | | | | |
| Participants | 13* | (a) Report numbers of individuals at each stage of study—eg numbers potentially eligible, examined for eligibility, confirmed eligible, included in the study, completing follow-up, and analysed | 8 | A total of 5697 dental students were recruited from 11 countries (Croatia, Iraq, Jordan, Kuwait, Lebanon, Malaysia, Nigeria, Saudi Arabia, South Africa, Turkey, and Yemen). |
|  |  | (b) Give reasons for non-participation at each stage |  | NA |
|  |  | (c) Consider use of a flow diagram |  | NA it is online-based questionnaire study and thus no missing data |
| Descriptive data | 14* | (a) Give characteristics of study participants (eg demographic, clinical, social) and information on exposures and potential confounders | 8 | The majority of the students were females (60.3%, n=3433), less than 20-years-old (67.6%, n=3853), and unmarried (94.1%, n=5361). Slightly more than half of the subjects (51.1%, n= 2909) were in the clinical years (Table 1). Also, 90.8% (n=5163) of the students reported that they had heard about E-cigarette, 1215 (23.6%) had tried E-cigarettes before, and 1872 (36.3%) reported that their family members/friends smoke E-cigarettes (Supplementary file, Table 2). |
|  |  | (b) Indicate number of participants with missing data for each variable of interest |  | NA |
|  |  | (c) *Cohort study*—Summarise follow-up time (eg, average and total amount) |  |  |
| Outcome data | 15* | *Cohort study*—Report numbers of outcome events or summary measures over time |  |  |
|  |  | *Case-control study—*Report numbers in each exposure category, or summary measures of exposure |  |  |
|  |  | *Cross-sectional study—*Report numbers of outcome events or summary measures | *8* | *The current status on the use of E-cigarette and/or tobacco smoking (N= 5676) are presented in Table 1. The majority of students (n= 4564, 80.4%) reported they never smoked, while 1112 (19.6%) students reported they were current smokers; of whom 596 (10.5 %) students smoke tobacco only, 255 (4.5%) students use E-cigarettes only, and 261 (4.7%) students were dual users. There were statistically significant gender-wise and country-wise differences regarding the use of E-cigarettes and/or tobacco cigarettes (P< 0.01). Meanwhile, no significant associations were found between smoking status and marital status, age, or level of study (Table 1).* |
| Main results | 16 | (*a*) Give unadjusted estimates and, if applicable, confounder-adjusted estimates and their precision (eg, 95% confidence interval). Make clear which confounders were adjusted for and why they were included | 9 | The proportions of students who gave correct responses to the different E-cigarettes knowledge questions ranged from 22.8% and 78.4% (Table 2). The majority (78.4%, n= 4035) of the students reported that E-cigarettes are harmful to health, 2160 (42%) reported that E-cigarettes pose a high risk for cancer, 2673 (51.9%) reported that E-cigarettes are addictive, 1148 (22.8%) reported that The FDA does not approve e-cigarettes, and 1833 (35.8%) reported that E-cigarettes do not reduce passive smoking. Interestingly, current E-cigarette users revealed poorer knowledge about E-cigarettes than non-smokers and tobacco cigarette users did (*P*< 0.01).  Around one-third (30.7%) of the students believed that E-cigarettes help in tobacco cessation. The majority (n= 4487, 87.2%) believed that dental practitioners should be educated about E-cigarettes, and 2451 (47.8%) agreed that E-cigarettes should be banned. In addition, 3929 (76.8%) students believed that education about E-cigarettes should start during the school years. Again, E-cigarette users significantly had more negative beliefs about E-cigarettes when compared to that of non-smokers and tobacco cigarette smokers (*P*< 0.01; Table 2). |
|  |  | (*b*) Report category boundaries when continuous variables were categorized |  | NA |
|  |  | (*c*) If relevant, consider translating estimates of relative risk into absolute risk for a meaningful time period |  | NA |

Continued on next page

| Other analyses | 17 | Report other analyses done—eg analyses of subgroups and interactions, and sensitivity analyses |  |  |
| --- | --- | --- | --- | --- |
| Discussion | | | | |
| Key results | 18 | Summarise key results with reference to study objectives | 11 | The present large-scale multicountry survey assessed the knowledge, beliefs, attitude, and practice of E-cigarette use among dental students across 11 countries. Generally, the results revealed that surveyed dental students have relatively positive beliefs and attitudes towards E-cigarette, but, unfortunately, unsatisfactory levels of knowledge with some significant differences across countries. Additionally, the results revealed that most students were non-smokers, while only 4.5% of students were E-cigarette users and 4.7% of them were dual users. |
| Limitations | 19 | Discuss limitations of the study, taking into account sources of potential bias or imprecision. Discuss both direction and magnitude of any potential bias | 15 | Despite being a multinational study, many limitations should be highlighted. First, the snowballing recruitment technique may have skewed the sample a bit if students shared it with friends who had similar demographics and lifestyles. A second limitation is the cross-sectional nature of the study and using a self-administered questionnaire, a tool that is criticized due to recall bias and subjectivity. Thirdly, as data were collected online, this may inadvertently had excluded students who did not have internet access. This excluded population may represent students with lower socioeconomic status, a matter which further may bias the obtained results. Fourthly, the included countries were composed of developed and developing countries, reflecting different cultures, and different education and health systems, a matter which also may contaminate the results and make the explanation challenging. Finally, the study did not explore the factors that prompt the use of tobacco and E-cigarettes by dental students; this may help to facilitate the design of programs to prevent or break the habit. |
| Interpretation | 20 | Give a cautious overall interpretation of results considering objectives, limitations, multiplicity of analyses, results from similar studies, and other relevant evidence | 15 |  |
| Generalisability | 21 | Discuss the generalisability (external validity) of the study results | NA |  |
| Other information | |  | | |
| Funding | 22 | Give the source of funding and the role of the funders for the present study and, if applicable, for the original study on which the present article is based | NA |  |

*Give information separately for cases and controls in case-control studies and, if applicable, for exposed and unexposed groups in cohort and cross-sectional studies.

**Note:** An Explanation and Elaboration article discusses each checklist item and gives methodological background and published examples of transparent reporting. The STROBE checklist is best used in conjunction with this article (freely available on the Web sites of PLoS Medicine at http://www.plosmedicine.org/, Annals of Internal Medicine at http://www.annals.org/, and Epidemiology at http://www.epidem.com/). Information on the STROBE Initiative is available at www.strobe-statement.org.
