# Supplementary material for: Knowledge, beliefs, attitude, and practices of E-cigarette use among dental students: A multinational survey
Source: PLoS One. 2022 Oct 27;17(10):e0276191. doi: 10.1371/journal.pone.0276191 (PMC9612543; doi:10.1371/journal.pone.0276191)
Supplement: S1 Questionnaire — (DOCX) [file pone.0276191.s002.docx]

**Knowledge, Attitude, Practice, and Beliefs about E-cigarettes/Vaping among Dental Students: A Multinational Comparison**

**Please respond to the following questions by placing a checkmark (√) in the answer that corresponds to your response**

| **What is your gender?** | ⃝ Male | | ⃝ Female | | |
| --- | --- | --- | --- | --- | --- |
| **How old are you?** | ⃝ ≤ 20 years | | ⃝ > 20 years | | |
| **Your Study level?** | ⃝ Pre-clinical | | ⃝ Clinical | | |
| **Current marital status?** | ⃝ Married | | ⃝ Unmarried | | |
| **Q1- Have you ever heard about e-cigarettes?** | | | | | |
|  | ⃝ Yes | | ⃝ No | | |
| **Q2- Have you ever personally tried e-cigarettes?** | | | | | |
|  | ⃝ Yes | | ⃝ No | | |
| **Q3- Do any of your close family members or close friends use e-cigarettes?** | | | | | |
|  | ⃝ Yes | | ⃝ No | | |
| **Q4- Do you currently use e-cigarettes or tobacco cigarettes?** | | | | | |
|  | ⃝ None, never smoke ⃝ Tobacco cigarettes only ⃝ E-cigarettes only ⃝ Dual user | | | | |
| IF YES, where did you learn about e-cigarettes? (Check everything that applies) | | | | | |
| ⃝ Dental school ⃝ Social media ⃝ Online advertising ⃝ Television/Radio advertisement ⃝ Public signs | | | | | |
| ⃝ Newspapers or Magazines ⃝ Others | | | | | |
| **Q5- Are e-cigarettes approved by the FDA for smoking cessation?** | | | | | |
|  | ⃝ Yes | | ⃝ No |  | ⃝ Don’t know |
| **Q6- E-cigarettes smoking is harmful to my health.** | | | | | |
|  | ⃝ Yes | | ⃝ No |  | ⃝ Don’t know |
| **Q7- The use of electronic cigarettes reduces the passive smoking of people around me.** | | | | | |
|  | ⃝ Yes | | ⃝ No |  | ⃝ Don’t know |
| **Q8- E-cigarettes are less harmful than tobacco cigarettes.** | | | | | |
|  | ⃝ Yes | | ⃝ No |  | ⃝ Don’t know |
| **Q9- Although the lack of long-term studies about the harmful effects of the e-cigarettes, they are a better option for my patients than smoking tobacco products.** | | | | | |
|  | ⃝ Yes | | ⃝ No |  | ⃝ Don’t know |
| **Q10- E-cigarettes are addictive.** | | | | | |
|  | ⃝ Yes | | ⃝ No |  | ⃝ Don’t know |
| **Q11- E-cigarettes pose a lower risk for cancer than traditional cigarettes?** | | | | | |
|  | ⃝ Yes | ⃝ No | |  | ⃝ Don’t know |
| **Q12- As a student, I feel confident about my ability to discuss the harmful effects of tobacco cigarettes use with my patients.** | | | | | |
|  | ⃝ Agree ⃝ Neutral ⃝ Disagree | | | | |
| **Q13- As a student, I feel confident about my ability to discuss the harmful effects e-cigarettes use with my patients.** | | | | | |
|  | ⃝ Agree ⃝ Neutral ⃝ Disagree | | | | |
| **Q14- Do you believe e-cigarettes are a helpful aid for smoking cessation?** | | | | | |
|  | ⃝ Agree ⃝ Neutral ⃝ Disagree | | | | |
| **Q15- Is it essential for a dentist to be educated about e-cigarettes?** | | | | | |
|  | ⃝ Agree ⃝ Neutral ⃝ Disagree | | | | |
| **Q16- Should e-cigarettes be banned?** | | | | | |
|  | ⃝ Agree ⃝ Neutral ⃝ Disagree | | | | |
| **Q17- What is, in your opinion, the best time to be educated about the harmful effects of e-cigarettes?** | | | | | |
|  | ⃝ At university ⃝ At school ⃝ No need | | | | |

| **Q18- How long have you been using e-cigarettes/tobacco cigarettes?** | | | | | |
| --- | --- | --- | --- | --- | --- |
| ⃝ < 1 year ⃝ 1–2 months ago ⃝ > 2 years ago | | | | | |
| **Q19- On average, how many times per day do you use e-cigarettes/tobacco cigarettes?** | | | | | |
|  | ⃝ Not-daily | ⃝ < 20 times a day | ⃝ ≥ 20 times a day |  | |
| **Q20- How soon after waking-up do you start using your e-cigarettes/tobacco cigarettes?** | | | | | |
| ⃝ Immediately after waking-up ⃝ After 1-2 hours ⃝ It varies | | | | | |
| **Q21- If I use e-cigarettes/tobacco cigarettes I will gain superiority among my friends?** | | | | | |
| ⃝ Agree ⃝ Neutral ⃝ Disagree | | | | | |
| **Q22- E-cigarettes/tobacco cigarettes give me more pleasure during its use?** | | | | | |
| ⃝ Agree ⃝ Neutral ⃝ Disagree | | | | | |
| **Q23- E-cigarettes/tobacco cigarettes relieve stress after using them?** | | | | | |
| ⃝ Agree ⃝ Neutral ⃝ Disagree | | | | | |
| **Q24- I want to quit e-cigarettes/tobacco cigarettes smoking?** | | | | | |
| ⃝ Agree ⃝ Neutral ⃝ Disagree | | | | | |
| **Q25- What was the reason for initiating e-cigarettes use?** | | | | | |
| 1- To protect my own health by reducing smoking or trying to quit smoking | | | | | |
| ⃝ Not important ⃝ Not sure ⃝ Important | | | | |  |
| 2- To protect my family members from secondhand smoke exposure | | | | | |
| ⃝ Not important ⃝ Not sure ⃝ Important | | | | |  |
| 3- To avoid smoking ban in public places | | | | | |
| ⃝ Not important ⃝ Not sure ⃝ Important | | | | |  |
| 4- Due to economic reasons (e-cigarettes are cheaper) | | | | | |
| ⃝ Not important ⃝ Not sure ⃝ Important | | | | |  |
| 5- To enjoy the various flavors of e-liquids | | | | | |
| ⃝ Not important ⃝ Not sure ⃝ Important | | | | |  |
