# Supplementary material for: Knowledge, beliefs, attitude, and practices of E-cigarette use among dental students: A multinational survey
Source: PLoS One. 2022 Oct 27;17(10):e0276191. doi: 10.1371/journal.pone.0276191 (PMC9612543; doi:10.1371/journal.pone.0276191)
Supplement: S1 Table — (DOCX) [file pone.0276191.s004.docx]

| S3 table: Scoring system of the study variables | | | |
| --- | --- | --- | --- |
| E-cigarettes are approved by FDA | Yes/Don’t know = 0 | No= 1 |  |
| E-cigarettes are harmful to my health | No/Don't know= 0 | Yes= 1 |  |
| E-cigarettes reduce passive smoking | Yes/Don’t know = 0 | No= 1 |  |
| E-cigarettes are less harmful than tobacco cigarette | Yes/Don’t know = 0 | No= 1 |  |
| Better option for my patients than tobacco products | Yes/Don’t know = 0 | No= 1 |  |
| E-cigarettes are addictive | No/Don't know= 0 | Yes= 1 |  |
| E-cigarettes pose a lower risk of cancer | Yes/Don’t know = 0 | No= 1 |  |
| **Knowledge score (higher score higher knowledge)** | **7 (range 0 - 7)** | |  |
|  | | | |
| E-cigarettes helps for smoke cessation | Disagree= 0 | Neutral= 1 | Agree= 2 |
| It is essential for a dentist to be educated about E-cigarettes | Disagree= 0 | Neutral= 1 | Agree= 2 |
| E-cigarettes should be banned | Disagree= 0 | Neutral= 1 | Agree= 2 |
| Time to be educated about harmful effects of E-cigarettes | No need= 0 | At university= 1 | At school= 2 |
| **Beliefs score (higher score good beliefs)** | **8 (range 0 - 8)** | | |
|  | | | |
| Feeling confident to discuss harmful effects of tobacco cigarettes | Disagree= 0 | Neutral= 1 | Agree= 2 |
| Feeling confident to discuss harmful effects of E-cigarettes | Disagree= 0 | Neutral= 1 | Agree= 2 |
| Superiority among friends | Agree= 0 | Neutral= 1 | Disagree= 2 |
| Feel more pleasure | Agree= 0 | Neutral= 1 | Disagree= 2 |
| Relieve stress | Agree= 0 | Neutral= 1 | Disagree= 2 |
| I want to quit smoking | Disagree= 0 | Neutral= 1 | Agree= 2 |
| **Attitude score (higher score positive attitude)** | **12 (range 0 - 12)** | | |
|  | | | |
| Duration of smoking | < 1 year= 1 | 1-2 years= 2 | > 2 years= 3 |
| Smoking per day | Not daily= 1 | < 20 times/day= 2 | ≥ 20 times/day= 3 |
| When start smoking | After wake-up= 1 | After 1-2 hours= 2 | It varies= 3 |
| **Practice score (higher score bad practice)** | **9 (range 3 - 9)** | | |
