# Supplementary material for: Knowledge, beliefs, attitude, and practices of E-cigarette use among dental students: A multinational survey
Source: PLoS One. 2022 Oct 27;17(10):e0276191. doi: 10.1371/journal.pone.0276191 (PMC9612543; doi:10.1371/journal.pone.0276191)
Supplement: S2 Table — (DOCX) [file pone.0276191.s005.docx]

| S4 table: Characteristics of participants by country (N= 5697) | | | | | | | | | | | |
| --- | --- | --- | --- | --- | --- | --- | --- | --- | --- | --- | --- |
|  |  | **Gender** | | **Age groups** | | **Educational level** | | **Marital status** | | **Heard about E-cigarette** | |
|  | **All** | **Male** | **Female** | **≤ 20 years** | **> 20 years** | **Pre-clinical** | **Clinical** | **Married** | **Unmarried** | **Yes** | **No** |
| All | 5697 (100.0) | 2264 (39.7) | 3433 (60.3) | 1844 (32.4) | 3853 (67.6) | 2788 (48.9) | 2909 (51.1) | 336 (5.9) | 5361 (94.1) | 5163 (90.8) | 526 (9.2) |
| Croatia | 233 (4.1) | 43 (18.5) | 190 (81.5) | 64 (27.5) | 169 (72.5) | 109 (46.8) | 124 (53.2) | 5 (2.1) | 228 (97.9) | 231 (99.1) | 2 (0.9) |
| Iraq | 369 (6.5) | 129 (35.0) | 240 (65.0) | 146 (39.6) | 223 (60.4) | 227 (61.5) | 142 (38.5) | 11 (3.0) | 358 (97.0) | 347 (94.0) | 22 (6.0) |
| Jordan | 461 (8.1) | 123 (26.7) | 338 (73.3) | 205 (44.5) | 256 (55.5) | 303 (65.7) | 158 (34.3) | 6 (1.3) | 455 (98.7) | 460 (99.8) | 1 (0.2) |
| Kuwait | 110 (1.9) | 12 (10.9) | 98 (89.1) | 25 (22.7) | 85 (77.3) | 39 (35.5) | 71 (64.5) | 4 (3.6) | 106 (96.4) | 109 (99.1) | 1 (0.9) |
| Lebanon | 257 (4.5) | 82 (31.9) | 175 (68.1) | 137 (53.3) | 120 (46.7) | 118 (45.9) | 139 (54.1) | 1 (0.4) | 256 (99.6) | 230 (89.5) | 27 (10.5) |
| Malaysia | 148 (2.6) | 35 (23.6) | 113 (76.4) | 38 (25.7) | 110 (74.3) | 32 (21.6) | 116 (78.4) | 0 (0.0) | 148 (100.0) | 144 (97.3) | 4 (2.7) |
| Nigeria | 240 (4.2) | 138 (57.5) | 102 (42.5) | 49 (20.4) | 191 (79.6) | 64 (26.7) | 176 (73.3) | 10 (4.2) | 230 (95.8) | 162 (67.5) | 78 (32.5) |
| Saudi Arabia | 596 (10.5) | 292 (49.0) | 304 (51.0) | 91 (15.3) | 505 (84.7) | 178 (29.9) | 418 (70.1) | 55 (9.2) | 541 (90.8) | 542 (90.9) | 54 (9.1) |
| South Africa | 204 (3.6) | 60 (29.4) | 144 (70.6) | 97 (47.5) | 107 (52.5) | 81 (39.7) | 123 (60.3) | 7 (3.4) | 197 (96.6) | 195 (95.6) | 9 (4.4) |
| Turkey | 1453 (25.5) | 695 (47.8) | 758 (52.2) | 640 (44.0) | 813 (56.0) | 710 (48.9) | 743 (51.1) | 33 (2.3) | 1420 (97.7) | 1353 (93.1) | 100 (6.9) |
| Yemen | 1626 (28.5) | 655 (40.3) | 971 (59.7) | 352 (21.6) | 1274 (78.4) | 927 (57.0) | 699 (43.0) | 204 (12.5) | 1422 (87.5) | 1390 (85.9) | 228 (14.1) |

| S4 table: Characteristics of participants by country (N= 5697) *continue* | | | | |
| --- | --- | --- | --- | --- |
|  | **Tried e-cig** | | **FF smoke e-cig** | |
|  | **Yes** | **No** | **Yes** | **No** |
| All heard about e-cig | 1215 (23.6) | 3929 (76.4) | 1872 (36.3) | 3284 (63.7) |
| Croatia | 68 (29.4) | 163 (70.6) | 85 (36.8) | 146 (63.2) |
| Iraq | 86 (24.8) | 261 (75.2) | 162 (46.7) | 185 (53.3) |
| Jordan | 170 (37.0) | 290 (63.0) | 292 (63.5) | 168 (36.5) |
| Kuwait | 11 (10.1) | 98 (89.9) | 57 (52.3) | 52 (47.7) |
| Lebanon | 48 (20.9) | 182 (79.1) | 96 (41.7) | 134 (58.3) |
| Malaysia | 17 (11.8) | 127 (88.2) | 50 (34.7) | 94 (65.3) |
| Nigeria | 12 (7.4) | 150 (92.6) | 30 (18.5) | 132 (81.5) |
| Saudi Arabia | 175 (33.3) | 350 (66.7) | 294 (54.2) | 248 (45.8) |
| South Africa | 91 (46.7) | 104 (53.3) | 110 (56.4) | 85 (43.6) |
| Turkey | 239 (17.7) | 1114 (82.3) | 197 (14.6) | 1156 (85.4) |
| Yemen | 298 (21.5) | 1090 (78.5) | 499 (36.1) | 884 (63.9) |
