# Supplementary material for: Knowledge, beliefs, attitude, and practices of E-cigarette use among dental students: A multinational survey
Source: PLoS One. 2022 Oct 27;17(10):e0276191. doi: 10.1371/journal.pone.0276191 (PMC9612543; doi:10.1371/journal.pone.0276191)
Supplement: S3 Table — (DOCX) [file pone.0276191.s006.docx]

| S5 table: Bivariate association between knowledge, beliefs and study variables | | | | |
| --- | --- | --- | --- | --- |
|  | **Knowledge (score= 0-7)** | | **Beliefs (score= 0-8)** | |
|  | **Mean±SD** | **P** | **Mean±SD** | **P** |
| All heard about e-cig | 2.9±1.7 |  | 5.8±1.3 |  |
| Country |  |  |  |  |
| Croatia | 2.6±1.5 | 0.000 | 5.6±1.1 | <0.001 |
| Iraq | 2.6±1.8 |  | 5.8±1.2 |  |
| Jordan | 2.8±1.7 |  | 6.2±1.0 |  |
| Kuwait | 2.9±1.7 |  | 6.2±0.9 |  |
| Lebanon | 2.5±1.6 |  | 5.7±1.1 |  |
| Malaysia | 3.7±1.7 |  | 5.9±1.0 |  |
| Nigeria | 2.4±1.9 |  | 6.0±1.0 |  |
| Saudi Arabia | 2.6±1.7 |  | 5.8±1.2 |  |
| South Africa | 2.7±1.7 |  | 5.8±1.0 |  |
| Turkey | 3.3±1.5 |  | 5.3±1.4 |  |
| Yemen | 2.8±1.9 |  | 5.9±1.3 |  |
| Gender |  |  |  |  |
| Male | 2.8±1.7 | 0.010 | 5.7±1.3 | <0.001 |
| Female | 2.9±1.7 |  | 5.8±1.2 |  |
| Age groups |  |  |  |  |
| ≤ 20 years | 2.9±1.7 | 0.935 | 5.7±1.3 | 0.360 |
| > 20 years | 2.9±1.8 |  | 5.8±1.2 |  |
| Training stage |  |  |  |  |
| Pre-clinical | 2.8±1.7 | 0.025 | 5.8±1.3 | 0.263 |
| Clinical | 2.9±1.7 |  | 5.7±1.2 |  |
| Marital status |  |  |  |  |
| Married | 2.8±1.8 | 0.232 | 5.9±1.2 | 0.012 |
| Unmarried | 2.9±1.7 |  | 5.7±1.3 |  |
| Tried E-cigarette |  |  |  |  |
| Yes | 2.5±1.6 | 0.000 | 5.7±1.3 | 0.009 |
| No | 3.0±1.8 |  | 5.8±1.2 |  |
| FF smoke e-cig |  |  |  |  |
| Yes | 2.7±1.7 | 0.000 | 5.8±1.3 | 0.009 |
| No | 3.0±1.8 |  | 5.7±1.3 |  |
| Currently smoker |  |  |  |  |
| Never smoke | 3.0±1.8 | 0.000 | 5.8±1.2 | <0.001 |
| Tobacco only | 2.8±1.7 |  | 5.4±1.4 |  |
| E-cig. only | 2.0±1.3 |  | 5.6±1.4 |  |
| Dual user | 2.4±1.5 |  | 5.4±1.4 |  |
