# Supplementary material for: Knowledge, beliefs, attitude, and practices of E-cigarette use among dental students: A multinational survey
Source: PLoS One. 2022 Oct 27;17(10):e0276191. doi: 10.1371/journal.pone.0276191 (PMC9612543; doi:10.1371/journal.pone.0276191)
Supplement: S4 Table — (DOCX) [file pone.0276191.s007.docx]

| S6 table: Bivariate association between attitude, practice and study variables | | | | |
| --- | --- | --- | --- | --- |
|  | **Attitude (score= 0-12)** | | **Practice (score= 1-9)** | |
|  | **Mean±SD** | **P** | **Mean±SD** | **P** |
| All smokers | 6.2±2.6 |  | 6.0±1.4 |  |
| Country |  |  |  |  |
| Croatia | 6.7±2.1 | 0.000 | 6.4±1.5 | 0.001 |
| Iraq | 6.8±2.4 |  | 6.3±1.3 |  |
| Jordan | 7.1±2.4 |  | 6.2±1.2 |  |
| Kuwait | 7.4±1.8 |  | 5.6±1.0 |  |
| Lebanon | 6.5±2.2 |  | 6.4±1.0 |  |
| Malaysia | 6.2±2.1 |  | 7.2±1.6 |  |
| Nigeria | 6.1±2.3 |  | 5.5±1.5 |  |
| Saudi Arabia | 6.8±2.7 |  | 6.1±1.3 |  |
| South Africa | 7.0±2.4 |  | 6.6±1.4 |  |
| Turkey | 5.0±2.3 |  | 5.7±1.5 |  |
| Yemen | 6.8±2.9 |  | 5.9±1.5 |  |
| Gender |  |  |  |  |
| Male | 6.4±2.5 | 0.004 | 6.1±1.5 | 0.001 |
| Female | 5.9±2.7 |  | 5.8±1.4 |  |
| Age groups |  |  |  |  |
| ≤ 20 years | 5.9±2.5 | 0.015 | 5.9±1.3 | 0.073 |
| > 20 years | 6.3±2.7 |  | 6.0±1.5 |  |
| Training stage |  |  |  |  |
| Pre-clinical | 6.2±2.6 | 0.658 | 6.0±1.3 | 0.220 |
| Clinical | 6.2±2.7 |  | 6.0±1.5 |  |
| Marital status |  |  |  |  |
| Married | 5.9±2.7 | 0.317 | 6.0±1.5 | 0.726 |
| Unmarried | 6.2±2.6 |  | 6.0±1.4 |  |
| Tried E-cigarette |  |  |  |  |
| Yes | 6.5±2.6 | 0.000 | 6.0±1.4 | 0.427 |
| No | 5.7±2.7 |  | 5.9±1.5 |  |
| FF smoke e-cig | | |  |  |
| Yes | 6.6±2.5 | 0.000 | 6.0±1.4 | 0.559 |
| No | 5.8±2.7 |  | 5.9±1.5 |  |
| Type of smoke |  |  |  |  |
| Tobacco only | 6.2±2.7 | 0.030 | 6.0±1.5 | 0.032 |
| E-cig. only | 6.5±2.6 |  | 5.9±1.3 |  |
| Dual user | 5.9±2.5 |  | 6.2±1.3 |  |
